# Food and Environment During the Late Roman Age at the Site of *Alba Fucens* (Abruzzi, Italy)

**DOI:** 10.3390/plants13202930

**Published:** 2024-10-19

**Authors:** Claudia Moricca, Gilda Russo, Duilio Iamonico, Emanuela Ceccaroni, Gabriele Favero, Laura Sadori

**Affiliations:** 1 Department of Environmental Biology, Sapienza University of Rome, Piazzale Aldo Moro 5, 00185 Rome, Italy; duilio.iamonico@uniroma1.it (D.I.); gabriele.favero@uniroma1.it (G.F.); laura.sadori@uniroma1.it (L.S.); 2 Department of Industrial and Information Engineering and Economics, University of L’Aquila, Via Giovanni Gronchi 18, 67100 L’Aquila, Italy; gilda.russo@guest.univaq.it; 3 Soprintendenza Archeologica, Belle Arti e Paesaggio per le Province di L’Aquila e Teramo, Via di San Basilio 2A, 67100 L’Aquila, Italy; emanuela.ceccaroni@cultura.gov.it

**Keywords:** Abruzzi, *Alba Fucens*, archaeobotany, Roman Age, waterlogging

## Abstract

Archaeobotanical analyses in Italy are uneven in terms of geographical and chronological distribution. Amongst the different regions, Abruzzi is poorly represented, with only one study covering the Roman Age. In this framework, the analyses carried out on carpological remains collected from the Late Roman (late 5th–early 6th century AD) filling of a well in the Sanctuary of Hercules in *Alba Fucens* represents an important addition to the state of the art. The plant assemblage consists of over 1500 remains attributed to 68 different taxa. These are partly represented by gathered fruit plants, such as *Corylus avellana*, *Juglans regia* and *Sambucus nigra*, while cereals and pulses are missing. An interesting aspect is represented by evergreen plants (*Pinus pinea* and *Cupressus sempervirens*) that are likely to have been used for ritual purposes rather than for human consumption. Finally, the impressive amount of ruderal and spontaneous plants represents a unicum for this type of study, allowing us to describe the past environment surrounding *Alba Fucens*, characterized by substantial water availability, Apennine grasslands and influenced by human presence.

## 1. Introduction

The region of Abruzzi (central Italy) represents a privileged case study for plant biodiversity due to its strategic position in the Mediterranan Basin, highly disrupted geomorphology, diverse lithology and climate [1]. In fact, this region has a high floristic richness, including 3566 *taxa* according to the most recent update of the Italian flora [2]. The landscape has been significantly modified by human actions since prehistory. A significant change occurred at the end of the 4th century BC, when the Romanization process began.

From an archaeobotanical point of view, Abruzzi is, after Valle d’Aosta, the second least investigated Italian Region, with data from only four sites published so far [3,4]. Of these, only one is of Roman age [5]. This research thus aims to enrich the state of the art by providing new data from the Roman site of *Alba Fucens* (currently Massa d’Albe municipality, L’Aquila administrative province, Abruzzi region), describing human-plant interactions, past environment, plant availability and diet. This study not only provides novel archaeobotanical information from a so far poorly studied region but is also crucial for understanding the way in which past populations adapted to a mountain environment. In fact, to this day, few studies have concerned Roman sites in Italy set at high altitudes [3,4], only two of which are found in central Italy [5,6].

### 1.1. The Site

**Figure 1:**
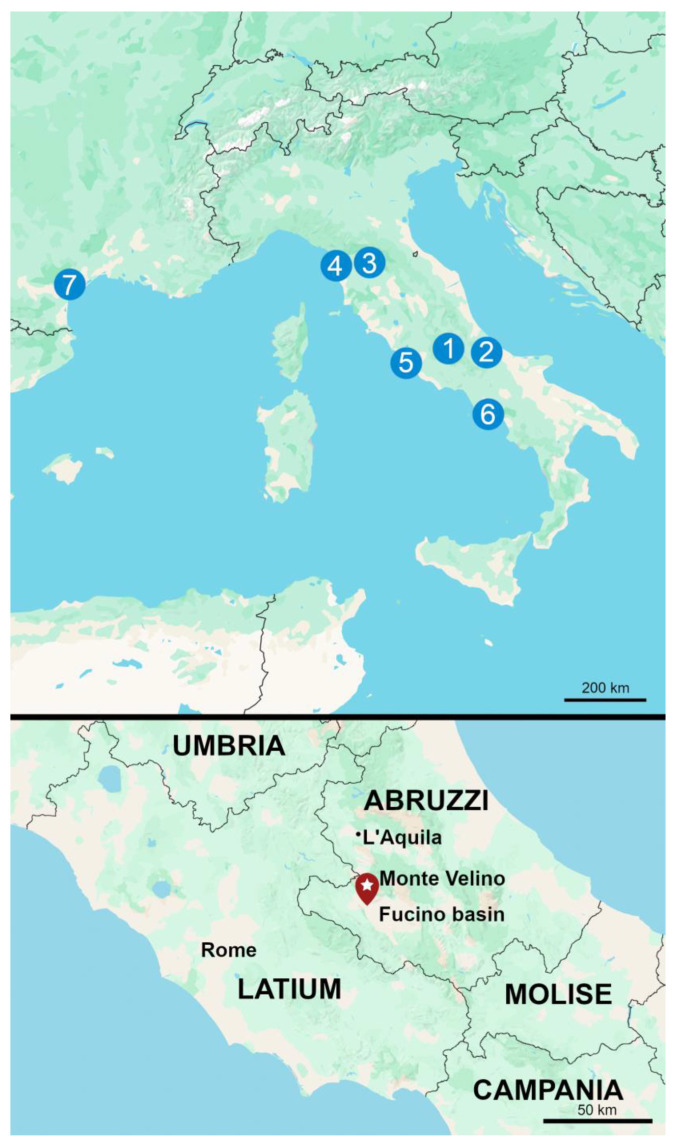
Geographical location of *Alba Fucens* (1 [top image; star [bottom image]) and of the other sites mentioned in the text: Acquachiara (2), Florence (3), Pisa (4), Portus (5), Pompeii (6), Béziers—Gasquinoy and La Lesse-Espagnac (7).

*Alba Fucens* (Massa d’Albe, L’Aquila, Abruzzi region) is set at an altitude between 949 and 990 m a.s.l., at the foothill of Mount Velino (Figure 1). It was mainly settled on Plio-Pleistocene clayey deposits and carbonate breccias [7]. The ancient city of *Alba Fucens* was founded at the end of the 4th century BC, within the framework of Rome’s expansion in central Italy. Its position was strategic due to the proximity of lake Fucino and the passage of the ancient road *Tiburtina Valeria*.

The history of the site intertwines with natural events, marked by a decline in late antiquity after centuries of prosperity. A devastating earthquake, believed to have struck in either 484 or 508 AD, significantly impacted the city’s later years, as attested by archaeological evidence [8]. Nonetheless, occupation of the area persisted also in the latest phases of antiquity, by exploiting remnants of the ancient city. Over time, sediment from repeated mass deposition events gradually filled the valley [7,9].

For two thousand years, the predominant economic activity throughout the region has been sheep farming, soon greatly supported by the Abruzzese practice of transhumance, which lasted until the mid-20th century [10,11]. Archaeological excavations of *Alba Fucens* began in 1949, by a Belgian team from the Catholic University of Leuven led by Fernand De Visscher, subsequently followed by Joseph Mertens until 1979, in collaboration with the Superintendence [12], which resumed the research in 2006.

### 1.2. The Well of the Sanctuary of Hercules

**Figure 2:**
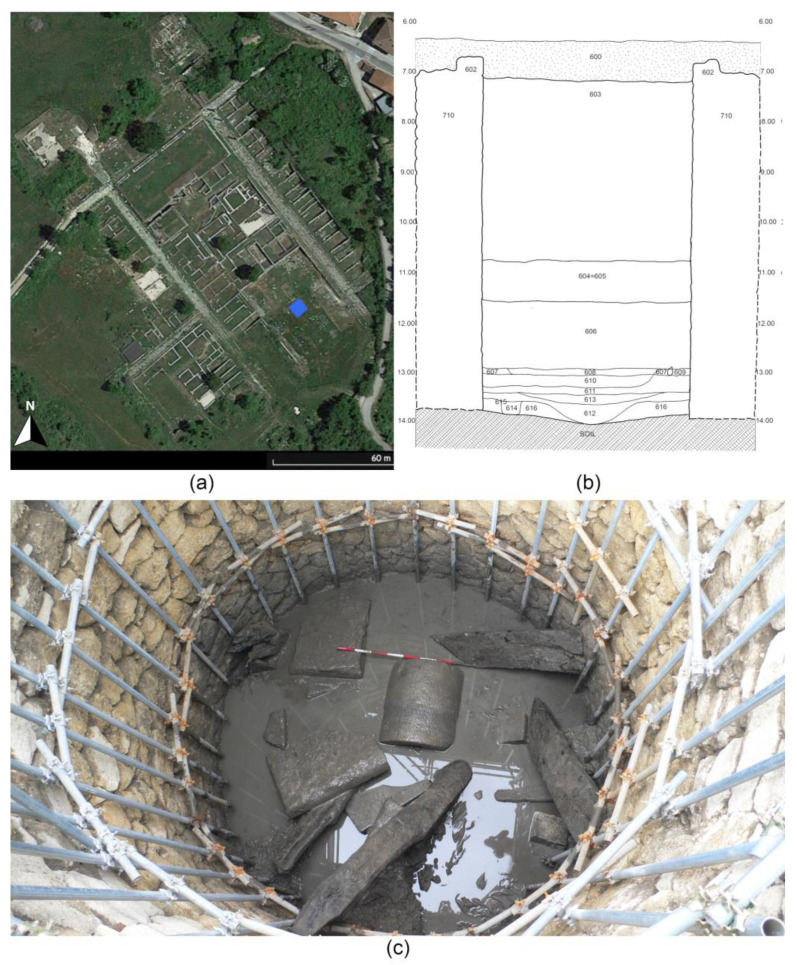
(**a**) Archaeological site of *Alba Fucens* and the Sanctuary of Hercules—the well is highlighted in blue; (**b**) stratigraphy of the well of the Sanctuary of Hercules – drawing by architect Paolo Fraticelli (reworked); (**c**) a picture of the well during the excavation.

Excavations led by the Superintendence between 2011 and 2014 have focused on a circular basin positioned directly beneath the layers of debris covering the yard of the shrine where the Hercules Epitrapezios statue was discovered. This basin, measuring ca. 4 m in diameter, has a depth of approximately 7 m (Figure 2). The connection of the well to other subterranean water passages allowed to identify it as part of a complex hydraulic system [13,14].

The structure of the well features polygonal masonry and has undergone multiple modifications at its upper edge. Below the top fill layer, primarily comprised of tiles and stone materials, fragments of columns, bases, and capitals were uncovered, constituting the final substantial sealing layer of the basin. Gradual removal of materials unveiled a deliberate accumulation of architectural elements, intermingled with various fragments of ceramics, glass, and marble. The specific damp environment facilitated the preservation of several wooden artifacts, including four lengthy beams still in situ, presumably forming part of the structure supporting the well. Sections of marble and bronze statues were also retrieved at various depths. The most recent filling of the well dates to the later period, specifically the late 5th–early 6th century AD, based on the presence of the latest pottery discovered. Most of the recovered items stem from the conclusive obliteration of the Hercules sanctuary, a consequence of the significant seismic event occurring just prior to 484 AD or in 508 AD. These effects reverberated even in Rome, evident from the documented damages noted in the paired inscriptions of the Colosseum [9]. The different stratigraphic units were difficult to distinguish based on their compositional and chromatic characteristics [13,14]. Overall, two main phases can be observed in the basin’s fill, related respectively to the use of the well (SUs 608-611; 613) and to its decommissioning (SUs 603-606). Finally, a clay layer (SU 612) intentionally placed to waterproof the bottom of the deposit, was identified at a depth of ca. 6.80 m. This features a depression and a circular pit about 50 cm in diameter (SU 615), located laterally and filled with small ceramic fragments (SU 614).

The contents of the well have already been studied in terms of faunal remains [15], wooden artifacts [16], ceramic [17] and stone materials [18], providing information about meat consumption, votive practices, and importation of raw materials.

## 2. Materials and Methods

Archaeobotanical analyses concerned six soil samples (total of 12.6 kg) from six different stratigraphic units (SU) of the filling of the well. The progressive numbering of the SUs indicates a greater depth with respect to the planking level. A blanket strategy with a judgmental element was adopted during the excavation of the context. A representative portion of each SU was sampled during the excavation for the study of plant macro-remains, in relation to the layer’s size. A control sample was kept for each SU. Considering the muddy nature and the limited size of SU 607, this layer was not sampled, as it was hard to collect sediment without risking contamination from nearby stratigraphic units. Instead, SU 614 was disregarded as mostly comprised of small ceramic fragments.

Separation of plant macro-remains was performed using a water sieving machine at the restoration laboratory of the Superintendence of Archaeology, Fine Arts and Landscape of Abruzzi, found at the *Museo delle Paludi* in Celano. Sediment was initially placed in buckets and covered with water, to favor the disaggregation of sediment. This was necessary as it had partially dried up during the years between the excavation and the archaeobotanical study, and dry sieving was not possible. Nonetheless, this does not appear to have damaged the recovered plant macro-remains. The broken-up sediment was then water sieved through meshes of size 5, 1 and 0.5 mm. This procedure was then followed by hand-picking of the remains.

Although it is preferable to preserve recovered materials waterlogged in similar conditions to those of recovery (to avoid distortion and destruction), long term storage may be problematic and require long-term monitoring [19]. Storing in water alone may induce the growth of micro-organisms, while the use of ethanol is incompatible with potential radiocarbon dating. For this reason, along with the necessity to transport samples to the Laboratory of Archaeobotany and Palynology of Sapienza University of Rome for identification, plant remains were slowly air-dried. This procedure does not appear to have damaged the recovered carpological material.

Identification of the separated remains was performed using a Zeiss Stemi 305 stereomicroscope (magnification 8–40×) and reference atlases [20,21]. Macro-remains were then photographed using a Leica M205C stereomicroscope and its incorporated Leica IC80 HD camera. They were subsequently processed using Helicon focus (version 6.6.1 Pro), which allows us to obtain images properly focused on the entire seed/fruit surface. In terms of quantification, entire individuals (seeds, achenes, cones, endocarps, mericarps, etc.) were separated from seed/fruit fragments (pericarp fragments, scales etc.). Botanical nomenclature follows the updated checklists of vascular Italian flora [22].

## 3. Results

A total of 1536 carpological remains belonging to 68 *taxa* were found in the studied deposit (Table S1). They represent both vascular plants and mosses. Plant remains are mostly attributable to gathered tree fruits (e.g., *Corylus avellana* L., *Juglans regia* L. and *Sambucus nigra* L.) and spontaneous herbaceous species (e.g., *Marrubium vulgare* L. and *Polygonum rurivagum* Jord. ex Boreau). Unfortunately, similarities between species belonging to the same families, identification at a species level was not always possible. A lower level of identification (family or genus level) was also reached for some of the remains fragmentation due to taphonomic processes or pre-depositional activities, not allowing to precisely assess the habitat of the recovered plant.

**Figure 3:**
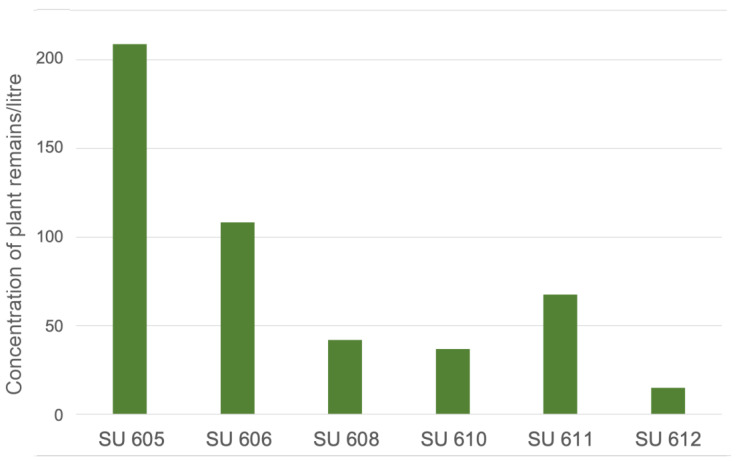
Concentration of plant remains in the studied stratigraphic units (SU). Stratigraphic units ordered from left to right according to the increasing depth of the well of the Sanctuary of Hercules.

All the studied materials were preserved by waterlogging, thanks to the presence of water in the well and its connection to other subterranean water passages. Out of the studied stratigraphic units, SU 605 (the most recent one) presents the highest concentration of plant remains (209 remains/kg), followed by SU 606 (108.2 remains/kg) and SU 611 (67.5 remains/kg) (Figure 3).

**Figure 4:**
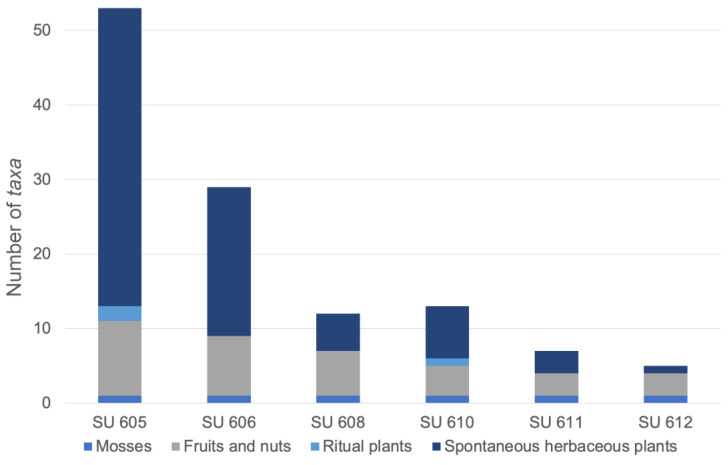
Number of *taxa* per stratigraphic unit (SU). Plant *taxa* are divided according to typology (please refer to Table S1 to see which *taxa* belong to each category).

A decrease of *taxa* diversity can be observed with the increasing depth of the well, with SU 605 featuring the highest diversity of remains (total of 53 *taxa*), followed by SU 606 (29 *taxa*), SU 610 (13 *taxa*), SU 608 (12 *taxa*), SU 611 (7 *taxa*) and SU 612 (5 *taxa*). The bottom layers, relative to the temple of Hercules’ functional period, are scant in plant remains, both related to spontaneous herbaceous plants and fruits/nuts (Figure 4). Identified *taxa* can be broadly divided into four categories: mosses, fruit and nuts, ritual plants and spontaneous herbaceous plants.

**Figure 5:**
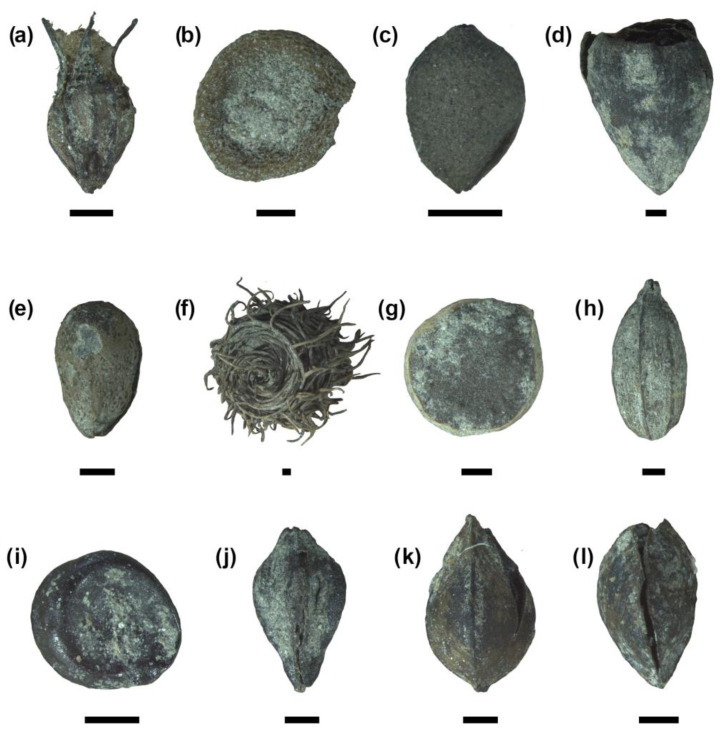
Weed seeds and fruits retrieved in the studied context (scale bar 0.5 mm): (**a**) *Armeria* cf. *arenaria*; (**b**) Brassicaceae; (**c**) *Carex* cf. *strigosa*; (**d**) *Carthamus* cf. *tinctorius*; (**e**) *Marrubium vulgare*; (**f**) *Medicago polymorpha*; (**g**) *Ranunculus arvensis*; (**h**) *Pimpinella major*; (**i**) *Chenopodium album*; (**j**) *Polygonum rurivagum*; (**k**) *Rumex crispus*; (**l**) *Rumex obtusifolius*.

Polygonaceae and Cyperaceae are the most represented families, with respectively 10 and 8 identified *taxa* each. Among them, the most abundant species are *Polygonum rurivagum* (Figure 5j) and *Carex* cf. *strigosa* (Figure 5c). Identified spontaneous herbaceous plants (Figure 5) nonetheless belong to numerous other families, including Apiaceae, Asteraceae, Caryophyllaceae, Lamiaceae and Ranunculaceae.

**Figure 6:**
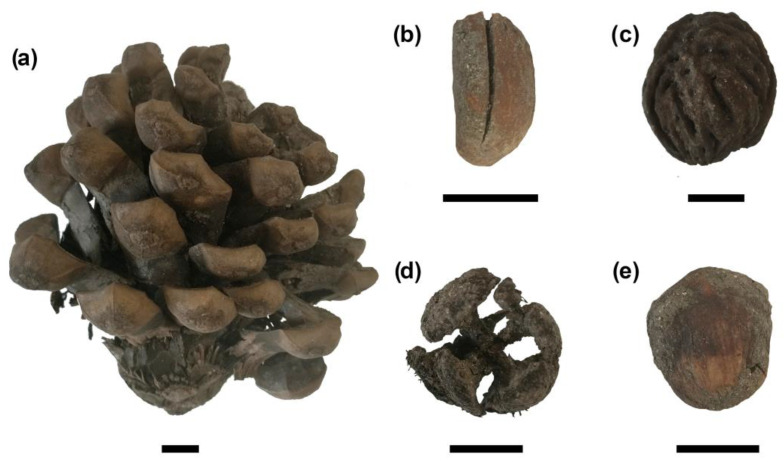
Archaeobotanical remains of arboreal plants retrieved from the studied well: (**a**) *Pinus pinea* cone; (**b**) *P. pinea* seed; (**c**) *Cupressus sempervirens* cone; (**d**) *Prunus persica* endocarp; (**e**) *Corylus avellana* pericarp.

Concerning gathered or cultivated plants, Caprifoliaceae remains are the most abundant, being represented by both *Sambucus nigra* (European elderberry) and *Sambucus ebulus* L. (dwarf elderberry). The presence of different tree nuts, including *Corylus avellana* (hazelnut) (Figure 6e), *Juglans regia* (walnut) and *Pinus pinea* L. (Mediterranean stone pine) (Figure 6a,b), is also interesting. Finally, fleshy fruits such as *Ficus carica* L., *Prunus avium* (L.) L.*/P. cerasus* L., *Prunus persica* (L.) Batsch (Figure 6c), *Rubus ulmifolius* Schott, and *Vitis vinifera* L. are also recorded in the archaeobotanical assemblage.

**Figure 7:**
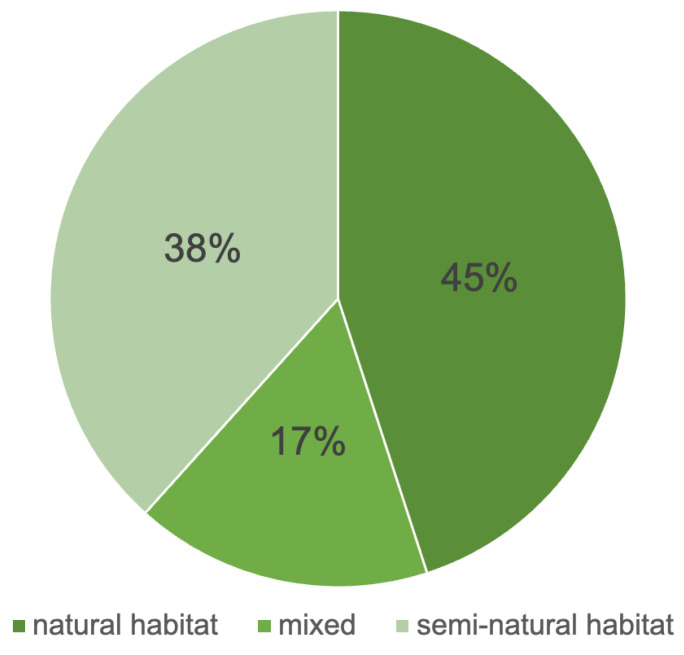
Habitats of plants recovered from the studied deposit. The term “mixed” is related to taxa that can occur both in natural environments and in environments affected by human presence. Percentages refer to the number of *taxa*.

In ecological terms, the identified *taxa* grow in a series of different habitats, that can be broadly divided into natural (45%—27 *taxa*), semi-natural habitats (38%—23 *taxa*) and a mix of the two (17%—10 *taxa*) (Table S1) (Figure 7). Natural habitats include woodlands, Mediterranean habitats, cliffs, humid grasslands and banks, wetlands, arid and rocky meadows. Instead, semi-natural habitats are those influenced by human presence such as urban and rural habitats, meadows and pastures, crops and walls.

## 4. Discussion

The present study is framed in a panorama of scarce archaeobotanical data in the Abruzzi region, thus representing an important step forward in the knowledge of past human-plant relationships in this specific geographic area. Furthermore, while the use of plants by ancient Romans has already been investigated in numerous sites around the Mediterranean basin (e.g., [23,24,25]), this paper highlights the way in which they adapted to a mountain environment.

### 4.1. Subsistence and Dietary Plants

Carpological remains of nine cultivated or gathered “fruit plants” were identified in the present study. These are represented by walnuts, hazelnuts, pine nuts, cherries, peaches, figs, grapes, blackberries and black elder. *Pinus pinea* is included in this category despite not being a fruit bearing plant, as its seeds are eaten by people. Most of these plants grow at elevated altitudes (>1000 m a.s.l.) and were likely available nearby the site [26]. *Ficus carica*, *P. pinea* and *Prunus persica* represent exceptions, most probably linked to human activities.

These data are partially coherent with those deriving from the site of Acquachiara in Abruzzi [5], where the recovered remains of fruit plants only concern grapes and figs. Nonetheless, the dietary *taxa* recorded in *Alba Fucens* are consistent with fruit and nut availability of ancient Romans. The presence of *Juglans regia* and *Vitis vinifera* is attested in most Roman sites of northern Italy, but *Corylus avellana*, *Ficus carica*, *Prunus avium/cerasus*, *Prunus persica*, *Rubus* sp., *Pinus pinea* and *Sambucus* ssp. are also attested in several sites [27]. Looking at central Italy, similar assemblages come, for example, from Florence [28] (here hazelnut, stone pine and elderberry were missing from the carpological record), Pisa [29] (*F. carica*, *V. vinifera* and *Sambucus* ssp. are not found) and Portus [23] (here records of walnut and stone pine are referred to wood). Eight out of the nine reported “fruit plants” are also recorded in the southern Italian site of Pompeii [24,30]. Assemblages containing the aforementioned plants are also recorded outside of the Italian peninsula (e.g., Gasquinoy, Southern France [25]).

The scarcity of *Sambucus nigra* records in central and southern Italy can be noticed when analyzing existing literature, especially if compared to its abundance at *Alba Fucens*. Although the black elder grows in a wide variety of habitats, being highly able to spread in anthropic environments, it prefers clearings, edges of damp woods and slopes [31]. Its abundance in *Alba Fucens*, along with that of *S. ebulus*, is likely related to the humid environments surrounding the site, also along the slopes of the city [32]. It is possible that black elderberry had culinary applications at *Alba Fucens*. In fact, *Apicius* mentions its use to make a savory pie which could be served eitherwarm or cold [33]. Furthermore, Roman treatises attest the use of elderberry for medicinal purposes, such as treating eye infections (in the form of a decoction of flowers and leaves) or skin diseases (directly applied on the skin in the form of poultices) [31]. Numerous remains of *Sambucus ebulus* were also found at *Alba Fucens*, suggesting that fruits of this plant were gathered [34]. As European elderberry, dwarf elderberry might have found a use for the treatment of numerous diseases and disorders [35]. Finally, both *Sambucus* species are recorded to have been used for dying fabrics and other products [31,36] thanks to the presence of tannins and other anthocyanins in their fruits [37].

Of particular interest is the retrieval of two peach endocarps in the studied sediment. They, along with numerous other endocarps handpicked from the well, represent the first finding of such species in the Abruzzi region. Native of western China, *Prunus persica* was cultivated in Persia, being later brought to Greece, and spreading to south-western Europe thanks to the Romans. Based on fossil evidence, its introduction to the Rome region, Latium, dates to the second half of the 1st century AD [38]. However, this could be related to scarce sampling for plant remains in Roman sites in Rome. In fact, according to the BRAIN database (Botanical Records of Archaeobotany Italian Network—brainplants.successoterra.net), only 11 Roman sites have been investigated in the capital from an archaeobotanical point of view [3]. Peach endocarp size is generally considered to be an index of cultivation, which led fruits to not only have larger and fleshier mesocarps, but also bigger endocarps [38]. Although two endocarps are not enough to draw a statistically significant hypothesis, some considerations can be made by comparing their size to that of other *Prunus persica* endocarps found during the Roman period in Italy. Their average measurements (length—25 mm; width—22.65 mm; thickness—17.3 mm) appear in line with those from the Colosseum west sewer (second half of the 4th century AD) [39], but bigger than the 1st century AD ones from *Privernum*, supporting the theory that a selection in peach tree cultivation occurred in Latium during the Imperial Age [38].

Another aspect to be discussed concerns the retrieval of a *Pinus pinea* cone and seed coat in the studied sediment. Worthy of mention is the presence of numerous other pinecones, collected by hand-picking during the excavation. While this plant presents a very low genetic variability along the entire Mediterranean basin, fossil evidence shows that it was present in the Iberian Peninsula for tens of thousands of years [40]. Its pre-Roman evidence in Italian archaeobotanical records is limited to Phoenician-Punic sites in insular Italy [41,42]. Romans played a key role in its diffusion [40]. Finally, the consumption of *Juglans regia* nuts in *Alba Fucens* also reflects the impact of the Romans, as this species spread significantly during Roman times [43].

All these archaeobotanical data complete the image of dietary habits of the late Roman inhabitants of the studied archaeological site. The study of faunal remains did, in fact, reveal a subsistence strategy heavily based on the consumption of meat of domesticated animals (mainly cattle, followed by swine), although wild specimens were also present. The use of ovine milk and dairy products is also heavily suggested by the presence of young individuals [15].

An aspect that appears to be unusual for the botanical assemblage of an archaeological site of Roman age is represented by the lack of cereal findings. It is likely that the lack of cereals is associated to the type of context and preservation. All plant remains related to arboreal fruit/nut plants in the deposit are comprised of woody endocarps and nut shells, which are not only inedible, but also more resistant to decay than fleshy parts. In numerous waterlogged contexts of Roman age [6,44], cereals are scarce and usually preserved by charring, probably being discarded as cooking by-products. Refuse from domestic activities is unlikely to have been deposited in the well of the Sanctuary of Hercules as it appears to have been used for ritual purposes.

The consumption of cereals at the site of *Alba Fucens* is hard to rule out, as also suggested by the presence of cereals in the nearby Roman site of Acquachiara (Abruzzi region) [5]. Nonetheless, Roman inhabitants of *Alba Fucens* could rely on imported cereals [45]. In fact, ancient sources, specifically the Latin poet *Silius Italicus* in his work *Punica*, mention the lack or scarcity of cereals in the proximity of the site, in favor of extensive orchards [46]. This may also be related to the heavy influence of lake Fucino on the territory of *Alba Fucens* over the centuries. The lack of a natural outlet caused its water level to fluctuate significantly, resulting in marshy shores and related diseases. For this reason, Romans designed an underground canal, whose construction started in 41 AD and became fully operational in the following century. After about three centuries, due to various complex reasons, the functioning of the canal progressively deteriorated, eventually allowing for the reformation of the ancient lake surface around the 6th century AD [47]. Humid conditions, also testified by several plant *taxa* present in the studied assemblage, are not favorable for cereal cultivation as most crops are sensitive to extreme environmental conditions such as waterlogging or submergence [48].

### 4.2. Ritual Plants

The ritual use of the well is supported by material evidence, which includes over a hundred wooden spinning tops, possibly referring to their votive significance. The offering of childhood toys, as part of rites of passage into adulthood, implies the surpassing of the previous condition. Other findings of votive offerings include numerous game pieces [16]. Interpretations of the use of the well can also be done in relation to its archaeobotanical assemblage. The bottom layers, relative to the temple of Hercules’ functional period, are scant in plant remains. Spontaneous herbaceous plants are mostly represented by aeolian remains, that is parts of plants are likely to have been deposited thanks to the action of wind. Evidence of gathered fruit plants in these layers is scarce, mostly represented by fig achenes, few nut fragments and single remains of grape, elderberry and peach. This is probably due to the well being part of a system for water collection and drainage rather than for disposal purposes. In contrast, the layers relative to the decommissioning of the well, are much richer in both edible plants and weeds, as these are likely to have been thrown in the well along with debris resulting from the destruction of the surrounding structures and may have included materials from the nearby altar of the temple.

While walnuts and hazelnuts, among others, are likely related to food consumption, the presence of *Cupressus sempervirens* (non-edible) and *Pinus pinea* cones could have another interpretation. Both cypress and stone pine were grown ornamentally in the Roman world [49,50,51]. Cypress was charged with mythological and religious symbolism by ancient Romans, that linked it to the funerary world and to the cult of Apollo. Ancient poets Ovid (43 BC—17 AD) and Virgil (70–19 BC) associated this tree to death and funerals in their writing [50], while Pliny (23–79 AD) reports a link between cypress and the Roman god Dis [51]. Written documentation concerning the ritual use of *P. pinea* cones comes from 3rd and 4th century AD Egyptian papyri, that mention them being designated for sacrificial purposes [49]. Furthermore, artistic and archaeological evidence of pinecones relates them with the cults of Mithras, Bacchus, Cybele and Silvanus [49]. Archaeobotanical evidence of both *P. pinea* and *C. sempervirens* is found in numerous ritual and funerary contexts throughout the Roman world (e.g., [29,52,53,54,55,56]). Particularly interesting is the finding of a *Cupressus* cone in a well (1st century BC—1st century AD) in the Roman site of La Lesse–Espagnac, where it could have been used as part of a foundation ritual [57]. The interpretation of cones as part of rituals in archaeological contexts is influenced by several factors. These include their position with respect to ritual structures. A charred state may indicate burnt offerings or the use as incense in rituals. It is also essential to compare the findings to the rest of the carpological assemblage they are part of. For example, a purposeful deposition is suggested by concentrated primary deposits of botanical remains which are not typically charred during food preparation, along with other distinctive artifact categories. Instead, the presence of numerous fragments of pine nutshells, along with cereal chaff or other crop-processing waste, implies that the assemblage is related to the preparation of foods [49]. The retrieval of pinecones in the well of the sanctuary of Hercules in *Alba Fucens* appears to support the hypothesis of ritual use, both due to the position within the temple and the type of assemblage, scarce in remains of food preparation and lacking cereals and pulses.

### 4.3. Environmental Indicators

Other than providing important information about the diet, subsistence and possibly ritual offerings of the inhabitants of *Alba Fucens* in the 5th century AD, the archaeobotanical assemblage is also useful to obtain information about the natural environment on the surrounding area.

Currently, the area of *Alba Fucens* is characterized by three main physiognomies, i.e., wall vegetation, shrubs, and meadows. The first mentioned type (also called “ruderal vegetation” *sensu* [58]) is characterized by a lesser floristic richness than those occurring in meadows due to various ecological factors, e.g., the thickness and moisture of the soil (not evoluted in walls; see e.g., [59]). Shrubs occur in *Alba Fucens* area as scattered formations, mostly dominated by taxa belonging to the family Rosaceae Juss. Finally, meadows which can be classified into two main types, mesophilous to semi-mesophilousm and therophytic dry grasslands. Mesophilous communities (dominated by *taxa* of Poaceae Barnhart) occupy most of the area of *Alba Fucens*, occurring among the ruins (amphitheatre and macellum) or other recent buildings (cementery and Saint Peter’s church) where soil is thick and well structured. On the other hand, therophytic dry grasslands (which include mainly annual herbs) are adapted to xero- and oligotrophic edaphic conditions [60] and can be found in specific zones of *Alba Fucens* area, e.g., the central oval plan of amphitheatre.

Spontaneous herbaceous plants in the carpological assemblage are represented mostly by Polygonaceae and Cyperaceae. Several *taxa* belonging to Polygonaceae commonly grow in anthropized areas, and so do other attested taxa, such as *Brassica* sp., *Medicago polymorpha* L., *Melissa officinalis* L., *Ranunculus arvensis* L., and *Poterium sanguisorba* L. Furthermore, *Chenopodium album* L. s.l., *Mercurialis annua* L., *Polygonum rurivagum*, *Rumex obtusifolius* L. s.l., and *Urtica dioica* L. are typical of ruderal areas.

The presence of pastures in the late Roman site of *Alba Fucens*, suitable for the millennial tradition of sheep herding, is attested by *Armeria* cf. *arenaria* (Pers.) F. Dietr. s.l., *Briza media* L. and *Marrubium vulgare*. This evidence is supported by numerous other taxa typical of meadows (e.g., *Cerastium* ssp., *Hypochaeris* sp., *Pimpinella major* (L.) Huds., and *Saponaria* sp.).

Carpological evidence attests an environment characterized by moist soils as those occurring near watercourses. Arboreal *taxa* described as being used as sources of food in Section 4.1 grow in mesophilic mixed woods (*Corylys avellana*, *Prunus avium/cerasus*), riparian woodlands (*Sambucus nigra*, *S. ebulus*) or edges of woods (*Juglans regia*). Numerous spontaneous herbaceous plants retrieved in the studied context also typically grow in humid soils, having a pioneer behavior in colonizing ruderal areas [31]. These include members of the well represented families Cyperaceae (*Carex* ssp., *Schoenoplectus* cf. *lacustris* (L.) Palla, and *Scirpus* sp.) and Polygonaceae (*Persicaria* sp.., *Rumex* cf. *hydrolapathum* Huds., and *Rumex* cf. *palustris* Sm.), but also other plants such as *Cirsium* cf. *arvense* (L.) Scop. and *Conium maculatum* L. Especially Cyperaceae (e.g., taxa of *Schoenoplectus*) include the so-called helophytes, i.e., semi-aquatic perennial plants with submerged perennating organs and with emergent stems; other taxa (e.g., several *Carex* species) are rhizomatose geophytes, i.e., herbaceous perennial plants bearing underground buds during the unfavourable season and which often growing in humid or acquatic habitats; finally other taxa (e.g., some members of Polygonaceae of the genera *Persicaria* or *Rumex*) are annual plants but ecologically strictly linked to water occurrence [61]. Therefore, the presence of these *taxa* in the area of *Alba Fucens* and their various biological adaptations, reveal a habitat characterized by a permanent of frequent occurrence of water (aquatic habitat), or regularly inundated (riparian habitat), or with soils water-embedded at least during a period of the year (humid habitat) [62,63]. The substantial water availability is, in fact, one of the reasons why the area was chosen as a settlement in the 4th century BC [64]. However, this implied that a massive remodeling was necessary for the reclamation of landscape. Soundings performed by the Superintendence of Archaeology of Abruzzi in 2012 attest the presence of stagnant waters during the 4th-3rd centuries BC. The natural landscape was heavily altered by the realization of terraces on the hill slopes and the implementation of a water disposal system.

As the mountain environment suggests, taxa associated to rocky substrates (e.g., *Iris* sp., *Saponaria* sp. and *Satureja* cf. *montana* L.) and cliffs (e.g., *Vincetoxicum* cf. *hirundinaria* Medik.) are also recorded.

Overall, the archaeobotanical assemblage describes a heavily anthropized environment, characterized by taxa typical of meadows and pastures, often growing on humid soils. Most identified species grow at altitudes up to 1400 m a.s.l. [65].

## 5. Conclusions

The present study has greatly allowed us to enrich the state of the art of archaeobotanical analyses in the Abruzzi region, also providing information about the subsistence of ancient Romans in Apennine areas. Other than being based on the consumption of meat, milk and dairy products, the diet of the inhabitants of the site included gathered and cultivated plants, which can be broadly divided into nuts and fleshy fruits. The impact of Romans on the availability of food plants is clear, with the presence of imported plants such as cypress, Mediterranean stone pine and peach. The latter is of particular interest, as the two endocarps retrieved represent the first *Prunus persica* findings in the Abruzzi region. The surprising lack of cereal remains in the assemblage is supported by the lack of signs of cereal processing at the site of *Alba Fucens* and ancient sources confirming the scarcity of cereals at the site in favor of extensive orchards. However, it appears more likely that cereals are missing due to the ritual nature of the context. Finally, based on the biological forms (*sensu* [61,66]) of the identified species (perennial helophytes or geophytes or some annuals), remains of herbaceous spontaneous plants allow us to identify the past environment surrounding *Alba Fucens*, characterized by substantial water availability, Apennine grasslands, and ruderal habitat influenced by human pressure.

## Data Availability

Data is contained within the article or Supplementary Material.

## Supplementary Materials

The following supporting information can be downloaded at: https://www.mdpi.com/article/10.3390/plants13202930/s1, Table S1: Carpological assemblage of the well of the Sanctuary of Hercules.
